# “Seeing” and “feeling” architecture: how bodily self-consciousness alters architectonic experience and affects the perception of interiors

**DOI:** 10.3389/fpsyg.2013.00354

**Published:** 2013-06-25

**Authors:** Isabella Pasqualini, Joan Llobera, Olaf Blanke

**Affiliations:** ^1^Atelier de la Conception de l'Espace, Institute of Architecture and the City, School of Architecture, Civil and Environmental Engineering, École Polytechnique Fédérale de LausanneLausanne, Switzerland; ^2^Laboratory of Cognitive Neuroscience, Brain Mind Institute, School of Life Sciences, École Polytechnique Fédérale de LausanneLausanne, Switzerland; ^3^Immersive Interaction Group, Faculté d'Informatique et Communications, École Polytechnique Fédérale de LausanneLausanne, Switzerland; ^4^Center of Neuroprosthetics, École Polytechnique Fédérale de LausanneLausanne, Switzerland; ^5^Department of Neurology, Geneva University HospitalGeneva, Switzerland

**Keywords:** bodily self-consciousness, embodiment, architecture, virtual reality, length estimation

## Abstract

Over the centuries architectural theory evolved several notions of embodiment, proposing in the nineteenth and twentieth century that architectonic experience is related to physiological responses of the observer. Recent advances in the cognitive neuroscience of embodiment (or bodily self-consciousness) enable empirical studies of architectonic embodiment. Here, we investigated how architecture modulates bodily self-consciousness by adapting a video-based virtual reality (VR) setup previously used to investigate visuo-tactile mechanisms of bodily self-consciousness. While standing in two different interiors, participants were filmed from behind and watched their own virtual body online on a head-mounted display (HMD). Visuo-tactile strokes were applied in synchronous or asynchronous mode to the participants and their virtual body. Two interiors were simulated in the laboratory by placing the sidewalls either far or near from the participants, generating a large and narrow room. We tested if bodily self-consciousness was differently modulated when participants were exposed to both rooms and whether these changes depend on visuo-tactile stimulation. We measured illusory touch, self-identification, and performed length estimations. Our data show that synchronous stroking of the physical and the virtual body induces illusory touch and self-identification with the virtual body, independent of room-size. Moreover, in the narrow room we observed weak feelings of illusory touch with the sidewalls and of approaching walls. These subjective changes were complemented by a stroking-dependent modulation of length estimation only in the narrow room with participants judging the room-size more accurately during conditions of illusory self-identification. We discuss our findings and previous notions of architectonic embodiment in the context of the cognitive neuroscience of bodily self-consciousness and propose an empirical framework grounded in architecture, cognitive neuroscience, and VR.

## Introduction

Inspired by Vitruvian theory, architects evolved several notions of embodiment (Vitruvius, 1st century BC). The classic ideal of architectonic embodiment, characterized as the bodily experience of a modular interior (e.g., as observed in the Pantheon in Rome), was further developed by Renaissance architects (Alberti, 1988). They proposed to enhance architectonic embodiment by combining modularity with experienced aspects of verticality (e.g., as known from the gothic domes) into a coherent architectonic form (Argan, 1946). Architectonic cues were thereby used to establish a sequence of predefined view and vanishing points in space, guiding the observer's body and eye movements through linear perspective according to the most advantageous effects of the architectonic forms. The thereby evoked impression of an architectonic continuum, comprehensive of modularity and verticality from a phenomenological point of view, has since then been referred to as the ideal of the Renaissance (Burckhardt, 1860).

Under the influence of the empirical biological sciences the notion of architectonic embodiment has been revisited in the nineteenth century, highlighting physiological and psychological aspects (Semper, 1860; Vischer, 1872; Lotze, 1884). Along these lines art historians have argued that the reference to the human body, by means of massive tectonic structures (i.e., crafted vertical elements) or spatial modules (i.e., regularly shaped void interiors) may facilitate the observer's identification with the environment through mechanisms of corporeal resonance (Wölfflin, 1886; Schmarsow, 1893). Particularly, Heinrich Wölfflin associated the physiognomy of the human body to architectonic cues based on oculomotor, somatosensory, and vestibular responses. Although the multisensory nature of architectonic embodiment is prominently present in Wölfflin's and others' theories, the exact role of own-body signals in the experience and appreciation of architecture has to date never been studied empirically.

In cognitive neuroscience embodiment and bodily self-consciousness have been associated with the feeling of owning a body, i.e., body ownership or self-identification (Tsakiris et al., 2007; Salomon et al., 2012), and the feeling of being located at one specific position in space, i.e., self-location (Schwabe and Blanke, 2008; Blanke and Metzinger, 2009). Studies in cognitive neuroscience have shown that self-identification and self-location can be modulated experimentally through visuo-tactile conflicts in healthy participants using immersive virtual reality (VR) to induce the so-called Full-Body-Illusion (FBI; Ehrsson, 2007; Lenggenhager et al., 2007; Petkova and Ehrsson, 2008; Aspell et al., 2009; Slater et al., 2009).

In the mentioned studies, participants wearing a head-mounted display (HMD) were filmed from behind at a distance of two meters and the filmed scene was projected on their HMD (Lenggenhager et al., 2007; Aspell et al., 2009). While participants were stroked with a rod on their backs, they were seeing on the HMD their own videotaped back or virtual body being stroked in front of them either in real-time, i.e., synchronous stroking, or with addition of a short delay, i.e., asynchronous stroking. In the synchronous condition participants reported illusory touch, that is, the feeling of being touched where they saw the virtual body being touched, and the feeling of self-identifying with the virtual body, that is, they felt as if the virtual body was their body (illusory self-identification). In addition, a change in self-location was found, meaning that participants showed a drift in self-location toward the virtual body. Both effects of self-identification and self-location were smaller or abolished for asynchronous stroking and also when the participants were presented a human-sized box as control object (Lenggenhager et al., 2007). Other studies have reported illusory ownership and self-identification with non-bodily objects in peripersonal space induced by visuo-tactile stroking (Armel and Ramachandran, 2003; Hohwy and Paton, 2010). Yet, how self-identification is influenced by visuo-spatial properties of the (architectonic) environment and vice versa has not been investigated previously.

Following principles of ecological psychology and embodied perception in experimental psychology, recent research has further revealed that own-body representations may influence how an observer perceives an environment (Gibson, 1979; Neisser, 1988). It has thus been demonstrated that perceived lengths in a visually presented environment might be influenced by the seen scale of one's own body within such an environment (Witt et al., 2005; Linkenauger et al., 2010). Moreover, such altered own-body perceptions seem to be modulated by experimentally induced changes in body ownership (van der Hoort et al., 2011). With respect to embodied perception in immersive VR it has also been shown that having a virtual representation of one's body in an immersive virtual environment helps to improve distance estimation (Mohler et al., 2010).

Here, we performed an experiment on the effects of two distinct architectonic interiors on bodily self-consciousness. We inquired whether experimentally induced illusory touch and self-identification (in the above described experimental setup; Lenggenhager et al., 2007) depends on the size of the architectonic interior by presenting the virtual body in a narrow or a large room and by exposing participants to synchronous and asynchronous visuo-tactile stimulation. To modulate the effect of room-size we placed two sidewalls next to the participants either in extrapersonal space, i.e., beyond reach (large room-size condition), or in peripersonal space, i.e., the space surrounding the body (narrow room-size condition; Fogassi et al., 1996; Rizzolatti et al., 1997; Ladavas et al., 1998; Teneggi et al., 2013). Additionally, we measured whether our participants' capacity to perceive the architectonic space dimensions changed during the different experimental conditions, expecting an improved ability during the illusion conditions. To this purpose we asked our participants to judge the length of several lines that we presented in the architectonic space during the different experimental conditions.

Our results show that synchronous stroking of the participant's body and the seen virtual body induces illusory touch and self-identification with the virtual body—independent from room-size. Furthermore, we found that weak feelings of illusory touch with the sidewalls and feelings of room retraction were induced in the narrow room. These subjective changes were complemented by a stroking-dependent modulation of length estimation only in the narrow room with participants judging the room dimensions more accurately during conditions of illusory self-identification. We discuss these data with respect to cognitive neuroscience of embodiment and bodily self-consciousness, and relate our data to theories of architectonic embodiment and VR.

## Materials and methods

### Participants

Twenty-four healthy, right-handed participants (5 females; 23.5 ± 2.8 years; mean age ± *SD*) participated in the study. All participants had normal or corrected to normal vision and no history of neurological or psychiatric conditions. Participants were sampled from the EPFL population. They were remunerated with 20 Sfr. an hour.

### Ethics statement

All participants gave written informed consent to the experiment. The study protocol was approved by the local ethics research committee—La Commission d'Ethique de la Recherche Clinique de la Faculté de Biologie et de Medecine at the University of Lausanne, Switzerland and was performed in accordance with the ethical standards laid down in the Declaration of Helsinki.

### Virtual reality setup

**Figure 1:**
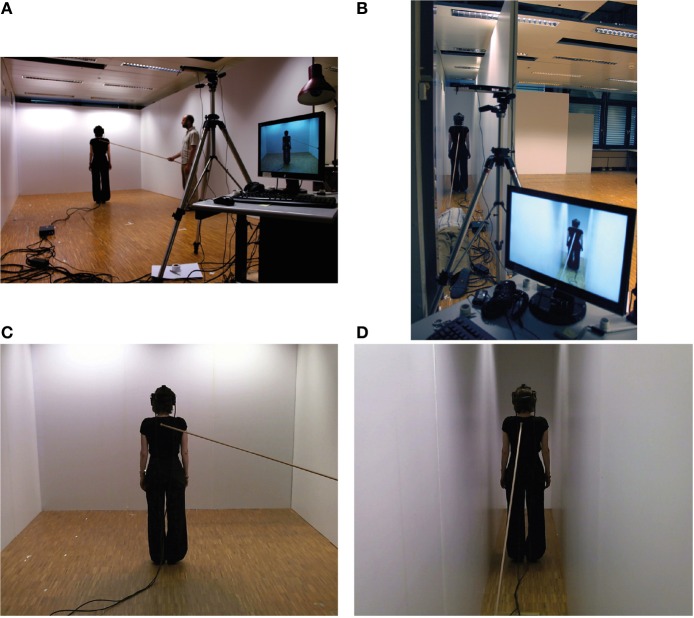
**The architectonic setup and the participant's view**. The setup with the movable walls and backstroking for the large room **(A)** and the narrow room **(B)**. The participant's impression of space viewed through the HMD for the large **(C)** and narrow room-sizes **(D)**. Filming of the scene in stereo 3D with two webcams combined to top lighting allowed a realistic representation of depth.

We used the video-based VR setup as previously described in Lenggenhager et al. (2007) and Aspell et al. (2009) and adapted it in such a way that the image seen by the participant would include a much larger proportion of space surrounding the virtual body (Figure 1). In the above-mentioned studies only the upper part of the participant's body was shown in a neutral environment and without any particular environmental cues. Also, the virtual body appeared to be closer to the participants' viewpoint, filling their visual field with the virtual representation of their own upper body. In our setup, however, the virtual body was entirely embedded in a virtual scene representing two distinct architectonic interiors, large and narrow, with the same room depth. The entire scene was filmed and dispatched in stereo, rendering the impression of a three-dimensional architectonic envelope that contained the fully embedded virtual body. For this purpose we used 2 webcams (Logitech C510, Apples, Switzerland) fixed on a tripod with 7 cm separation using a custom support to generate a stereo image, projected on a high definition Head-Mounted-Display (HMD, model Virtual Research Systems VR1280, Aptos, CA), rendering 2 images of 1280 × 1024 pixels at 60 Hz, with a diagonal field of view of 60°. The cameras were centered on the backside of either room, aligned at the same distance and directed toward the front wall.

In order to change the size of the room (factor *room-size*) in which both, the subject and the virtual body were standing, we used 4 custom-made panels (1.50 × 2.50 meters, length × height) mounted on wheels, aligned as one 6 meters long movable wall (Figures 1A,B). In combination with one corner of the experimentation room the movable walls formed either a large room (surface 6 × 3.50 meters, length × width) or a narrow room (surface 6 × 0.80 meters, length × width). Synchronous or asynchronous stroking (factor *stroking*) was applied to the back of the participants by the use of a wooden stick as described previously (Figures 1C,D) (Lenggenhager et al., 2007). For synchronous stroking the image captured from the webcams was directly relayed to the HMD. For asynchronous stroking a delay (800 ms) was inserted.

**Figure 2:**
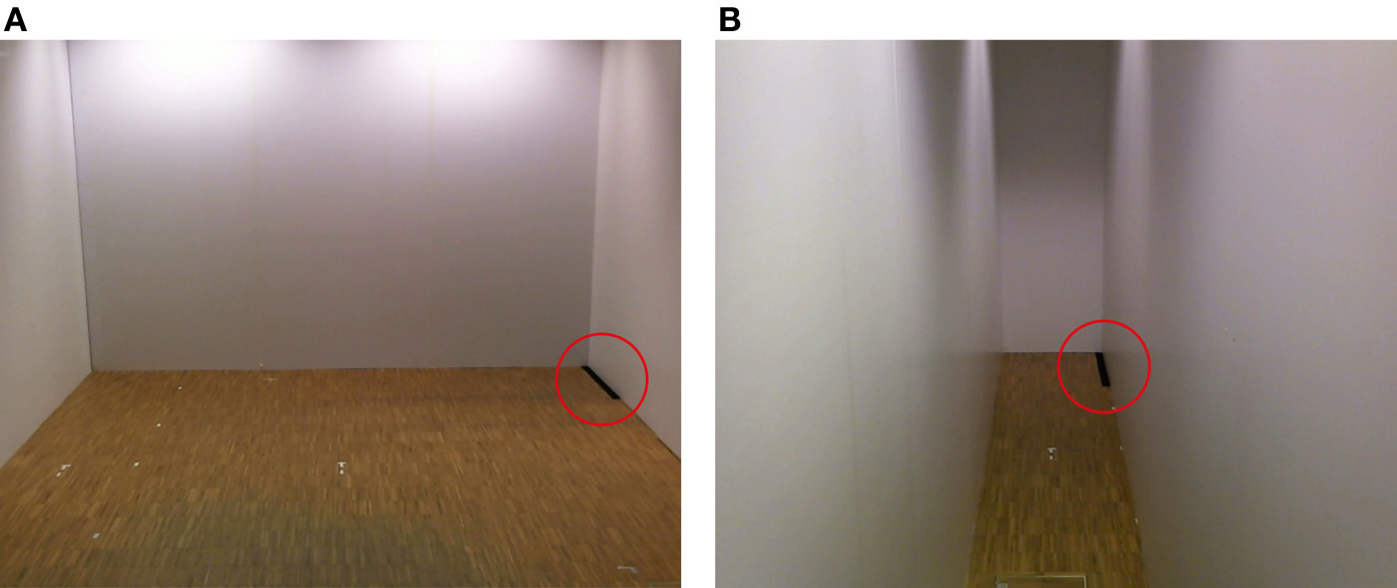
**Stimuli for length estimation presented in perspective**. The stimuli of the length estimation task (black bars) as presented on the HMD after each block of the FBI for the two interiors. The bars were presented in perspective along the sidewalls of the large **(A)** and narrow room **(B)**.

Length estimation was tested with previously captured images of the two interiors. The images showed the same setup from the identical visual angle, yet, including some black bars presented in different positions and orientations (Figure 2). A vertical black bar (50 cm) placed at the bottom center of the white front wall was used as the reference bar for the estimations. The bars involved in the length estimation consisted of: 5 horizontal bars placed on the bottom of the front wall (lengths of 40, 45, 50, 55, and 60 cm); 5 bars seen on the ground in perspective and centered with respect to the room-size (lengths 40, 50, 60, 70, and 80 cm); 5 bars seen on the ground along the right wall (lengths 40, 50, 60, 70, and 80 cm; Figure 2); and 5 bars seen on the ground along the left wall of lengths 40, 50, 60, 70, and 80 cm. For each of the four experimental conditions we presented a total of 20 bars in randomized order.

### Procedure

Participants were asked to wear the HMD for the whole duration of each experimental condition and to stand at the center of the room (large or narrow) facing the front wall at a distance of 4.5 meters from the webcams (Figure 1). Before carrying out the experimental conditions we performed a training session, allowing the study participants to familiarize with the setup. The vertical reference bar was presented to the participants, followed by one shorter and one longer horizontal bar. For each subject the cameras and the HMD were calibrated and adjusted ensuring a correct visual angle throughout the four conditions.

The four experimental conditions were run in separate blocks with randomization of factors: *room-size* (large/narrow) and *stroking* (synchronous/asynchronous) in a full 2 × 2 factorial design. Participants were filmed from behind by the webcams and watched, on the HMD, their body standing in front of them as a virtual body (Figures 1C,D). First, participants were exposed to the reference bar and then to visuo-tactile stroking for 2 min. Then participants were asked to perform a two-alternative forced choice task, responding whether they perceived the presented bars as longer or shorter than the reference bar (Figure 2). Responses were collected by means of a joystick (Logitech Cordless RumblePad 2). During the four experimental conditions the participants were exposed to white noise over the HMD headphones. ExpyVR (http://lnco.epfl.ch/expyvr) software was used to render the stimuli and record the responses. Size estimation was carried out immediately after the 2 min stroking period. At the end of each experimental condition, the HMD was removed and participants were requested to answer a 14 item written questionnaire and to rate each item on a Likert scale between −3 (not at all) and +3 (very much) (Table 1).

**Table 1:** **The questionnaire completed after each experimental block**.

**“During the past moments sometimes…”:**	
(1) …	I was feeling the touch of the stick where i saw the virtual body being touched.
(2) …	I clearly felt that the stick touching the virtual body was causing the touch I was feeling.
(3) …	I clearly felt that the virtual body was my body.
(4) …	It seemed as if my physical body was drifting toward the virtual body.
(5) …	It seemed as if I might have more than one body.
(6) …	It seemed as if I was standing in two places at the same time.
(7) …	I felt as if the walls of the room were almost lightly touching me.
(8) …	I had the impression to see the front wall drifting toward me.
(9) …	It seemed as if I was floating in the room.
(10) …	I could feel that I was standing inside the room.
(11) …	I felt as if the void space was becoming a part of myself.
(12) …	I felt as if the walls were getting closer to myself.
(13) …	I felt that the virtual environment was a place, rather than an image.
(14) …	The first time I saw the virtual body disappear I was pulled into the space.

### Data analysis

Data analysis was performed with Matlab R2012a (Mathworks Inc., Natick, MA) and Statistica 10 (Statsoft Inc., Tulsa, OK). Questionnaire scores were analyzed using repeated-measures analyses of variance (ANOVA) with the factors *stroking* (synchronous/asynchronous), *room-size* (large/narrow), and *question* (1–14; Table 1) as within-participant factors. For further *post-hoc* tests for specific significant interactions we performed *t*-tests. In order to avoid false positives we excluded any significant answer that was not confirmed if tested with an alternative and more critical non-parametric test (Wilcoxon test).

The analysis of performance in the length estimation task was done using Probit analysis (Craven, 1993; Howe and Purves, 2002, 2005). Probit analysis provides a method to determine the error distribution in the length estimation of bars of different lengths presented in a task in comparison to a reference bar. The experimental factors were introduced as possible indicators of the variations of estimation. Since Probit analysis is a particular type of generalized linear model, we normalized all the experimental factors to introduce the Beta values as indicators of the relative strength of the different factors. A factor was considered to be relevant, if it showed a significance level of *p* < 0.05. In addition, the factor was only included if it resulted in a significantly smaller residual error of the fit (χ^2^ comparison test, *p* < 0.05).

To the best of our knowledge Probit analysis has not been used previously in experiments addressing bodily self-consciousness. Therefore, as a complementary analysis on the same data we considered the subjective length estimations (of each particular bar length and position) in relation to the probability threshold of perceiving the bars as shorter than the reference bar. For example, a probability threshold of 0.5 corresponds to a bar length subjectively estimated as being equal to the length of the reference stimulus [for example see Kannape et al. (2010) for a similar measure]. We excluded those participants, whose subjective estimation thresholds were outside the range of estimation of the stimuli presented for any chosen threshold. A pilot study revealed the tendency to underestimate the length of the presented stimuli. Hence, in order to include a greater amount of participants, we favored a 0.75 probability threshold of answering shorter, instead of a 0.50 probability threshold of equal length (ruled out by the forced-choice task). This was analyzed using repeated measures ANOVA and Bonferroni *post-hoc* tests.

## Results

### Questionnaire responses

Questionnaire analysis revealed the following two-way interactions: *question* by *room-size* [Partial η^2^ = 0.165, *F*_(13, 299)_ = 4.54, *p* < 0.001] and *question* by *stroking* [Partial η^2^ = 0.428, *F*_(13, 299)_ = 17.22, *p* < 0.001; Table 1]. We also found the following main effects: *room-size* [Partial η^2^ = 0.190, *F*_(1, 23)_ = 5.4069, *p* = 0.0292], *stroking* [Partial η^2^ = 0.555, *F*_(1, 23)_ = 28.72, *p* < 0.001] and *question* [Partial η^2^ = 0.535, *F*_(13, 299)_ = 26.47, *p* < 0.001]. No other effects were significant.

**Figure 3:**
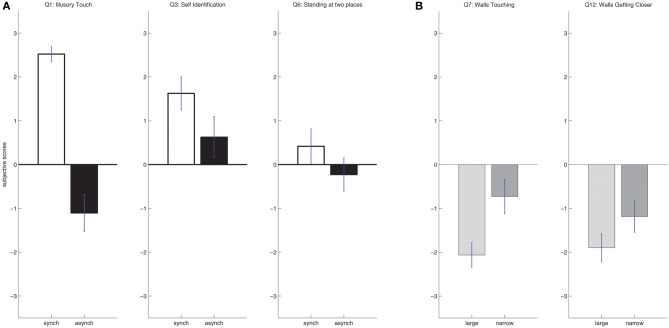
**Subjective responses. (A)** Bodily self-consciousness. Results of the questionnaire responses are shown (mean and standard error) for the items that depended on *stroking* (questions 1 and 3). **(B)** Architectonic embodiment. Results of the questionnaire responses are shown (mean and standard error) for the items that depended on *room-size* (questions 7 and 12).

Further *post-hoc* testing of the *question* by *stroking* interaction revealed a significant variation with *stroking* for three questions (Figure 3A; Table 1). These were question 1 [… I was feeling the touch of the stick where I saw the virtual body being touched; *t*_(1, 23)_ = 9.3134, *p* < 0.001], where the synchronous condition scored higher (mean = 2.52, s.e. = 0.18) than the asynchronous condition (mean = −1.10, s.e. = 0.42); question 2 [… I clearly felt that the stick touching the virtual body was causing the touch I was feeling; *t*_(1, 23)_ = 5.6168, *p* < 0.001], where again the synchronous condition revealed a higher score (mean = 2.02, s.e. = 0.36) than the asynchronous condition (mean = −0.86, s.e. = 0.46) and question 3 [… I clearly felt that the virtual body was my body; *t*_(1, 23)_ = 2.6602, *p* = 0.014], where the synchronous condition also showed higher scores (mean = 1.62, s.e. = 0.39) than the asynchronous (mean = 0.62, s.e. = 0.47). None of the control questions was significantly modulated by factor *stroking*.

Further *post-hoc* testing with respect to the *question* by *room-size* interaction showed a significant variation for question 7 and 12 (Figure 3B; Table 1). Question 7 referred to illusory touch with the architectonic interior (… I felt as if the walls of the room were almost lightly touching me). Although ratings were low we found question 7 to be significantly stronger [*t*_(1, 23)_ = 3.5973, *p* = 0.002] in the narrow (mean = −0.73, s.e. = 0.39) as compared to the large room condition (mean = −2.06, s.e. = 0.29). Ratings for question 12 were also low and inquired about room retraction (… I felt as if the walls were getting closer to myself). This was found to be significantly stronger [*t*_(1, 23)_ = 3.1759, *p* = 0.030] in the narrow room (mean = −1.2, s.e. = 0.35) than in the large room (mean = −1.90, s.e. = 0.32). No further question was modulated by factor *room-size*.

### Length estimation

**Figure 4:**
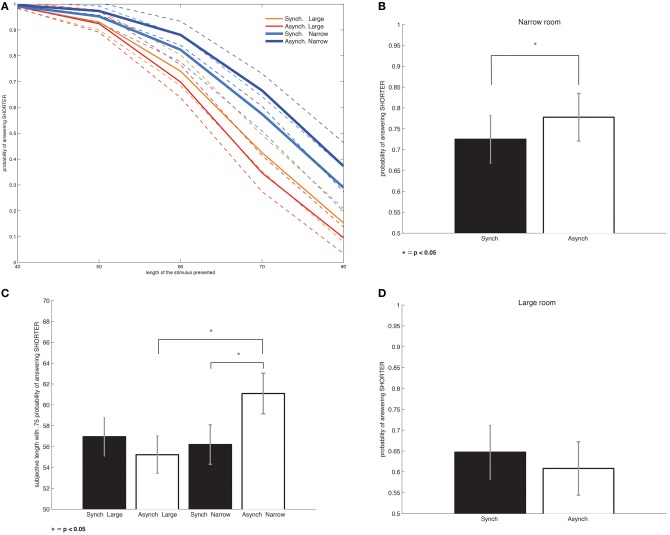
**Bodily self-consciousness modulates length estimation. (A)** The result of the Probit analysis shown for the four conditions across all bar lengths. The fit of the curves together with the confidence intervals suggest that the modulation in length estimation is specific for the narrow room and modulated by the *stroking* synchrony factor. **(B)** The effect of the *stroking* synchrony factor on the responses, independent of the presented bar length, is shown considering only responses in the narrow room. A Probit analysis shows a significant effect of the *stroking* synchrony factor, irrespective of the bar length. **(C)** The same Probit analysis considering only responses from the large room shows no significant effect of the *stroking* synchrony factor, **(D)** An analysis of variance of the subjective bar length corresponding to a 0.75 probability threshold shows an interaction between the *stroking* synchrony factor and *room size*. Bonferroni *post-hoc* tests show significant differences between synchronous and asynchronous *stroking* only in the narrow room. There are also differences between the large and the narrow room, but only for asynchronous *stroking*. ^*^p < 0.05

Several factors significantly contributed to the probability of perceiving the bars shorter than the reference bar (Figure 4A). As expected the main factor was the length of the presented bars (beta = −1.154, *p* < 0.001). The factor *room-size* (beta = −0.2785, *p* < 0.001) and the interaction between *stroking* and *room-size* were also significant (beta = 0.1017, *p* = 0.018). No other factors, such as the bar positions, affected the responses.

In order to further analyze the *stroking* by *room-size* interaction we repeated the Probit analysis considering the large and narrow room-size conditions separately. This analysis revealed that the synchronous narrow room condition was the main driving factor for the interaction (beta = −0.1209, *p* = 0.049; Figure 4B). The same analysis for the large room condition was not significant when considered separately (beta = 0.0827, *p* = 0.170; Figure 4C), suggesting that stroking synchrony only affected length estimations when participants performed the estimations in the narrow room.

The same results were found in a complementary analysis using repeated measures ANOVA on the subjective bar length estimation thresholds. The subjective threshold of 0.75 probability of answering shorter included 19 of 24 participants (see the methods section for the exclusion criterion). The interaction *room-size* by *stroking* was significant [Partial η^2^ = 0.318, *F*_(1, 18)_ = 8.389, *p* = 0.01]. Bonferroni *post-hoc* tests revealed significant differences between large and narrow room-size conditions for asynchronous stroking (*p* = 0.011). *Stroking* was also a significant factor, yet only in the narrow room-size condition (*p* = 0.043; see Figure 4D). Overall, ANOVA and Bonferroni *post-hoc* tests confirmed the interaction factor found in the Probit analysis for the subjective length at a 0.75 probability level.

The length estimations for the horizontal bars positioned along the front wall showed a different behavior. The length of the bars was revealed to be the main factor (*p* < 0.001). Stroking and the interaction stroking by room-size were not found to be significant (but revealed a non-significant trend; *p* = 0.08). No other significant factors were found.

The data on length estimation show that across all conditions participants estimated the bars presented in perspective as shorter than the target line. However, in the narrow room, length estimation significantly improved when participants self-identified with the virtual body (synchronous narrow condition), compared to when self-identification was not present (asynchronous narrow condition). This effect did not occur for the large room.

## Discussion

Our data show that synchronous stroking of the participant's body and the seen virtual body induces illusory touch and self-identification with the virtual body. Self-identification and illusory touch were not differently modulated by the two different room-sizes. On the other hand, weak feelings of illusory touch with the sidewalls and the feeling of the approaching walls (room retraction) were induced by the narrow room-size. These subjective changes were complemented by a stroking-dependent modulation of length estimation only in the narrow room with participants judging the room dimensions more accurately during conditions of illusory self-identification.

### Architectonic experience and visuo-tactile bimodality

The FBI is characterized by self-identification with the virtual body and a measured drift in self-location toward the virtual body. In our experimental setup a FBI was induced through visuo-tactile conflicts between *felt* touches applied to the participants' back and *seen* touches applied to the back of a virtual body. Participants were filmed from behind and their image was dispatched on their HMD, as previously tested by Lenggenhager et al. (2007) and Aspell et al. (2009). Our results confirm the outcome of the mentioned studies with respect to illusory touch and self-identification. In the questionnaires we did not find these aspects of bodily self-consciousness to be directly modulated by the two different *room sizes*.

Asking participants to judge their experiences of the interiors after the FBI, we found that they experienced very mild sensations of being touched by the sidewalls as well as the feeling that the sidewalls were drifting toward them (retraction). Both sensations were stroking-independent and differed for both room sizes, being stronger in the narrow room-size condition. This finding may suggest a mild effect of embodiment of the walls (touch) and of containment (experienced retraction of the sidewalls) induced by the *room-size*.

Previous studies between a participant's stroked physical hidden hand and seen strokes applied to a tabletop or non-human object induced feelings of illusory touch for the table *by stroking* (Armel and Ramachandran, 2003; Hohwy and Paton, 2010). Describing a touch illusion induced through visuo-tactile stroking of a white box and a hidden participant's hand, Hohwy and Paton (2010) found that the physical hand could be felt to extend toward the box. It was argued that the participants probably experienced the hand to be located inside the box and therefore the touch was felt as if “through the box.” In the questionnaire responses of our study, participants did not declare such mediating role of *stroking* with respect to the entire body, nor the whole interior. We argue that the present illusion of feeling touched by the sidewalls could only be induced visually through the architectonic elements located in peripersonal space, and without any dependence on visuo-tactile stroking as observed by others for a table or box (Armel and Ramachandran, 2003; Hohwy and Paton, 2010).

These visually induced touch sensations in conditions with closer walls seem to relate to previous findings about increased stimulus detection in peripersonal space (Rizzolatti et al., 1981). Several studies have since revealed visuo-tactile integration, especially when in close proximity to the body of the observer (Ladavas et al., 1998; Haggard et al., 2007; Sambo and Forster, 2009; Teneggi et al., 2013). We thus speculate that the narrow room, which was perceived within peripersonal space (10 cm or less lateral distance from participants' physical body), may be embodied visually and associated with mild feelings of touch and containment (room retraction). This effect was absent when the architectonic boundaries were outside peripersonal space, that is, in more distant extrapersonal space. Visuo-spatial perception in the large room might therefore be related to distinct perceptual and bodily mechanisms, such as visual perception linked to oculomotor processes (Witt and Proffitt, 2007). With respect to self-location in an interior space, this result suggests that bodily self-consciousness is extended toward those architectonic elements that are located in close proximity to the physical body.

### Visuo-spatial estimation depends on the experience of space

We introduced a behavioral measure that would allow testing the participants' ability to perceive the depth of the room, in function of the FBI. In our hypothesis we assumed that an increased ability of length estimation in three-dimensional space would correlate with the participants' experience of having their center of awareness inside the virtual room and body, i.e. through self-location and self-identification. Indeed, biases in the length estimation task were significantly modulated by the experimental conditions and associated with changes in self-identification and room-size experience. We also note that, consistent with previous work on length estimation, changes in length estimation affected all tested lengths in the same fashion (Craven, 1993; Howe and Purves, 2002, 2005).

The estimations were modulated in a stroking- and room-size-dependent fashion, as the interaction between *stroking* and *room-size* revealed. We expected that *stroking* would improve length estimations by increasing the sensation of spatial depth through multisensory perception of the virtual interior (associated with conditions of higher self-identification with the virtual body). Compared to the narrow room, in the large room length estimations were generally improved yet independently from *stroking* (i.e., synchronous vs. asynchronous stroking did not affect length estimations in the large room).

A possible interpretation of the difference between the large and the narrow interior may be given by the visual scaling of the virtual body inside the changed room proportions, as has already been argued before (Witt et al., 2005; Linkenauger et al., 2010). Such scaling-related visuo-spatial perception has also been shown to be related to self-identification with virtual space and body (van der Hoort et al., 2011). In addition, self-identification with the virtual body may have also induced a transposition of the center of perspective toward the virtual interior: in the narrow room this may have led participants to perceive the otherwise underestimated length more realistically, as it has been shown that self-identification with an avatar can reduce HMD-induced space-compression (Mohler et al., 2010). Since in the large room the FBI did not affect length estimations, we propose that architectonic elements close to the body increased the effect of spatial embodiment in combination to self-identification with the virtual body.

These findings are in line with investigations of embodied perception and notions of ecological psychology, stating that vision depends in a fundamental way on the experience of the body in space (Gibson, 1979; Neisser, 1988). Our results indicate that self-identification with the virtual body during the FBI may have mentally displaced the observer into the architectonic space when mediated through a visual stimulus in peripersonal space. This suggests that self-identification can be extended to architectonic elements close to the body in contrast to a human-sized object (box) shown in extra-personal space (Lenggenhager et al., 2007). These findings are compatible not only with a somatosensory impact of architecture through visual stimulation (as tested here in Question 7 and 12), but also with improved visuo-spatial perception (length estimation) due to increased self-identification with elements of the apparently retracting room allowing at the same time for a more vivid and realistic experience of those portions of the interior perceived in extra-personal space. Further studies could reveal if these measurable changes in spatial perception may be associated with self-location and an experienced displacement into the virtual interior.

### Architecture from the first-person perspective

Linear perspective, if applied to immersive VR, allows to visually mimic the observer's first-person perspective by encoding subjective bodily parameters as viewpoint (body size and location), vanishing point (gaze direction), and monocular cues (i.e., size, line convergence, foreshortening, occlusion, texture, luminance, motion, and aerial perspective; Gibson, 1979; Cutting, 1997). In our VR setup the only cues to determine the relative dimension of room-size, were the virtual body filmed by the distanced camera in combination with perspective convergence in the narrow room, or, the visibility of the front wall in the horizontal direction in the large room. In the narrow condition the front wall was partly occluded by the virtual body (Figures 1C,D) and the sidewalls were more convergent toward the virtual body. In the large room condition, the virtual body and the front wall represented the most important spatial cues.

For both rooms, we did not observe any significant improvement of length estimation due to *stroking* for the bars presented horizontally on the front wall. This may be due to the higher visibility of the bars, allowing a more direct comparison with the reference bar. Accordingly, the present results in length estimation seem also to be compatible with the reported forward displacement in self-location that is absent along the right-left axis (Lenggenhager et al., 2007; Aspell et al., 2009). Thus, Lenggenhager et al. (2007) and Lenggenhager et al. (2009) found changes in the forward (or sagittal) direction, but not toward the right or the left. In the present setup the sagittal direction of the effects generally associated with the FBI also seems to be present in length estimation in the narrow room and only influence the latter in the sagittal direction.

### Architectonic and bodily space

Interior space conveys a perceptual experience that is fundamental for architecture. Since ancient times this experience has been related to the phenomenology of space assumedly occurring through embodiment of a structural mass or spatial modules (Vitruvius, 1st century BC; Alberti, 1450; Semper, 1860). Crucial to the theories of architectonic embodiment is therefore the phenomenological transposition of the experience of architectonic space toward the masses or voids, hence translating own-body perception into architectonic dimensions. The outcome of our study may be discussed with respect to Heinrich Wölfflin's and August Schmarsow's notions of embodiment. In Heinrich Wölfflin's theory tectonic expressivity (i.e., achieved through crafted elements) facilitates embodiment with respect to the architectonic environment (Wölfflin, 1886). The arousal of characteristic architectonic sensations (e.g., familiarity, safety, containment, infinity, etc.) and “moods” (e.g., atmospheres) seems therefore to be evoked through an empathic resonance that the built environment induces within the human body. Hereby the structural moments of architectonic articulation can be felt by empathic observers, related to bodily physiognomy (e.g., through orientation, verticality, and symmetry) and to own-body processing.

Early theories of empathy (or sympathetic projection; Vischer, 1872; Lotze, 1884) mention that oculomotor mechanisms induce a bodily resonance within form and space, particularly through a transposition of perspective toward the figurative mass. In Wölfflin's interpretation, architectonic experience is not only linked to oculomotor (and potentially vestibular mechanisms), but architectonic form also resonates with mechanisms related to the bodily boundaries, in terms of a haptic (i.e., tactile, proprioceptive, and motor) response to visual and vestibular stimuli (Wölfflin, 1886, 1915). Crucially, stressing links to bodily processing, Wölfflin mentions that architectonic asymmetry would have comparable effects on the human body “as if a limb was missing” (Wölfflin, 1886).

August Schmarsow's reply to this proposition points beyond Vischer's and Wölfflin's visuo-motor and physiological mechanisms (Schmarsow, 1893). He speculates about a human sense of space historically developed in the interiors of primordial inhabitations and related to somesthetic projections. In his terms, the sense of space is based on the somesthetic experience of the architectonic interiors and on an “objectified” perspective gained by a perspective transposed toward the interior void. Schmarsow's observer can thus be characterized as being positioned inside an architectonic void, experiencing the interior from *within* (and not from *in front* as the observer in Wölfflin's theory). Furthermore, through self-displacements within the void, the sense of space seems to bear an objectified notion, through which previous points of self-location may be identified from several locations, thus progressively generating higher-level perspective representations through space.

By highlighting aspects of bodily self-consciousness in the perception of architectonic space, including motor aspects, both, Wölfflin and Schmarsow, highlighted experiences related to bodily processing for the understanding and the appreciations of architecture. Our empirical data strengthen this link and indicate a potential relation between architectonic theory and bodily self-consciousness (Blanke and Metzinger, 2009). The present participants experienced differences in illusory touch and room retraction depending on *room-size*, suggestive of a mild self-identification not only with the virtual body, but also with the walls through an involvement of somatosensory mechanisms, compatible with suggestions by Wölfflin and Schmarsow.

Our behavioral data show that in the narrow room such architectonic embodiment, probably modulated through the sidewalls, concerned not only the tectonic elements (walls) but the whole architectonic interior, as the length stimuli presented in the direction of the perspective of our participants were more realistically estimated in the FBI. Illusory touch with the sidewalls may be in line with Wölfflin's notion of resonance, whereas self-identification with the virtual interior in combination with improved size estimation after the FBI in the narrow room might relate to a projection of bodily space toward the architectonic void, and, of the bodily boundaries toward the enclosure. Schmarsow's observer—being positioned *within* the architecture and experiencing the space from a first-person perspective oriented toward the void—could therefore be related to experimentally induced changes of bodily self-consciousness of the observer through the FBI in immersive VR.

### Conflict of interest statement

The authors declare that the research was conducted in the absence of any commercial or financial relationships that could be construed as a potential conflict of interest.

## References

[B1] Alberti L. B. (1450). De re Aedificatoria (On the Art of Building in Ten Books). Cambridge: The MIT Press (1988).

[B2] Argan G. C. (1946). The architecture of brunelleschi and the origins of perspective theory in the fifteenth century. J. Warburg Courtauld Inst. 9, 99–121 10.2307/750311

[B3] Armel K. Ramachandran V. (2003). Projecting sensations to external objects: evidence from skin conductance response. Proc. Biol. Sci. 270, 1499–1506 10.1098/rspb.2003.2364 12965016 PMC1691405

[B5] Aspell J. E. Lenggenhager B. Blanke O. (2009). Keeping in touch with one's self: multisensory mechanisms of self-consciousness. PLoS ONE 4:e6488 10.1371/journal.pone.0006488 19654862 PMC2715165

[B6] Blanke O. Metzinger T. (2009). Full-body illusions and minimal phenomenal selfhood. Trends Cogn. Sci. 13, 7–13 10.1016/j.tics.2008.10.003 19058991

[B7] Burckhardt J. (1860). The Civilization of the Renaissance in Italy. London: Middlemore (1878).

[B8] Craven B. J. (1993). Orientation dependence of human line-length judgments matches statistical structure in real-world scenes. Proc. Biol. Sci. 253, 101–106 10.1098/rspb.1993.0087 8396771

[B9] Cutting J. E. (1997). How the eye measures reality and virtual reality. Behav. Res. Methods Instrum. Comput. 29, 27–36 10.3758/BF03200563

[B10] Ehrsson H. H. (2007). The experimental induction of out-of-body experiences. Science 317, 1048 10.1126/science.1142175 17717177

[B11] Fogassi L. Gallese V. Fadiga L. Luppino G. Matelli M. Rizzolatti G. (1996). Coding of peripersonal space in inferior premotor cortex (area F4). J. Neurophysiol. 76, 141–157 8836215 10.1152/jn.1996.76.1.141

[B12] Gibson J. J. (1979). The Ecological Approach to Visual Perception. New York, NY: Psychology Press, Taylor and Francis (1986).

[B13] Haggard P. Christakou A. Serino A. (2007). Viewing the body modulates tactile receptive fields. Exp. Brain Res. 180, 187–193 10.1007/s00221-007-0971-7 17508208

[B14] Hohwy J. Paton B. (2010). Explaining away the body: experiences of supernaturally caused touch and touch on non-hand objects within the rubber hand illusion. PLoS ONE 5:e9416 10.1371/journal.pone.0009416 20195378 PMC2827559

[B15] Howe C. Q. Purves D. (2002). Range image statistics can explain the anomalous perception of length. Proc. Natl. Acad. Sci. U.S.A. 99, 13184–13188 10.1073/pnas.162474299 12237401 PMC130607

[B16] Howe C. Q. Purves D. (2005). Natural-scene geometry predicts the perception of angles and line orientation. Proc. Natl. Acad. Sci. U.S.A. 102, 1228–1233 10.1073/pnas.0409311102 15657143 PMC544621

[B18] Kannape O. A. Schwabe L. Tadi T. Blanke O. (2010). The limits of agency in walking humans. Neuropsychologia 48, 1628–1636 10.1016/j.neuropsychologia.2010.02.005 20144893

[B19] Ladavas E. Di Pellegrino G. Farne A. Zeloni G. (1998). Neuropsychological evidence of an integrated visuotactile representation of peripersonal space in humans. J. Cogn. Neurosci. 10, 581–589 10.1162/089892998562988 9802991

[B20] Lenggenhager B. Mouthon M. Blanke O. (2009). Spatial aspects of bodily self-consciousness. Conscious. Cogn. 18, 110–117 10.1016/j.concog.2008.11.003 19109039

[B21] Lenggenhager B. Tadi T. Metzinger T. Blanke O. (2007). Video ergo sum: manipulating bodily self-consciousness. Science 317, 1096–1099 10.1126/science.1143439 17717189

[B22] Linkenauger S. A. Ramenzoni V. Proffitt D. R. (2010). Illusory shrinkage and growth: body-based rescaling affects the perception of size. Psychol. Sci. 21, 1318–1325 10.1177/0956797610380700 20729479 PMC3302719

[B23] Lotze H. (1884). Grundzüge der Aesthetik. Leipzig: Hirzel

[B24] Mohler B. J. Creem-Regehr S. H. Thompson W. B. Bulthoff H. H. (2010). The effect of viewing a self-avatar on distance judgments in an HMD-based virtual environment. Presence Teleoper. Virtual Environ. 19, 230–242 10.1162/pres.19.3.230

[B25] Neisser U. (1988). Five kinds of self-knowledge. Philos. Psychol. 1, 35–59 10.1080/09515088808572924

[B26] Petkova V. I. Ehrsson H. H. (2008). If I were you: perceptual illusion of body swapping. PLoS ONE 3:e3832 10.1371/journal.pone.0003832 19050755 PMC2585011

[B27] Rizzolatti G. Fadiga L. Fogassi L. Gallese V. (1997). The space around us. Science 277, 190–191 10.1126/science.277.5323.190 9235632

[B28] Rizzolatti G. Scandolara C. Matelli M. Gentilucci M. (1981). Afferent properties of periarcuate neurons in macaque monkeys. II. Visual responses. Behav. Brain Res. 2, 147–163 10.1016/0166-4328(81)90053-X 7248055

[B29] Salomon R. Van Elk M. Aspell J. Blanke O. (2012). I feel who I see: visual body identity affects visual–tactile integration in peripersonal space. Conscious. Cogn. 21, 1355–1364 10.1016/j.concog.2012.06.012 22832215

[B30] Sambo C. F. Forster B. (2009). An ERP investigation on visuotactile interactions in peripersonal and extrapersonal space: evidence for the spatial rule. J. Cogn. Neurosci. 21, 1550–1559 10.1162/jocn.2009.21109 18767919

[B31] Schmarsow A. (1893). The Essence of Architectural Creation, in Empathy, Form and Space–Problems in German Aesthetics 1873–1893, ed Mallgrave H. F. (Santa Monica, CA: The Getty Center for the History of Art and the Humanities, 1994), 125–148

[B32] Schwabe L. Blanke O. (2008). The vestibular component in out-of-body experiences: a computational approach. Front. Hum. Neurosci. 2:17 10.3389/neuro.09.017.2008 19115017 PMC2610253

[B33] Semper G. (1860). Style in the Technical and Tectonic Arts; or, Practical Aesthetics. Los Angeles, CA: The Getty Research Institute, (2004).

[B34] Slater M. Perez-Marcos D. Ehrsson H. H. Sanchez-Vives M. V. (2009). Inducing illusory ownership of a virtual body. Front. Neurosci. 3, 214–220 10.3389/neuro.01.029.2009 20011144 PMC2751618

[B35a] Teneggi C. Canzoneri E. di Pellegrino G. Serino A. (2013). Social modulation of peripersonal space boundaries. Curr. Biol. 23, 406–411 10.1016/j.cub.2013.01.043 23394831

[B35] Tsakiris M. Hesse M. D. Boy C. Haggard P. Fink G. R. (2007). Neural signatures of body ownership: a sensory network for bodily self-consciousness. Cereb. Cortex 17, 2235–2244 10.1093/cercor/bhl131 17138596

[B36] van der Hoort B. Guterstam A. Ehrsson H. H. (2011). Being Barbie: the size of one's own body determines the perceived size of the world. PLoS ONE 6:e20195 10.1371/journal.pone.0020195 21633503 PMC3102093

[B37] Vischer R. (1872). On the optical sense of form: a contribution to aesthetics, in Empathy, Form and Space–Problems in German Aesthetics 1873–1893, ed Mallgrave H. F. (Santa Monica, CA: The Getty Center for the History of Art and the Humanities, 1994), 89–123

[B38] Vitruvius M. P. (1st century BC). De Architectura Libri Decem (Zehn Bücher über Architektur). Darmstadt: Wissenschaftliche Buchgesellschaft (1891, 2008).

[B39] Witt J. K. Proffitt D. R. (2007). Perceived slant: a dissociation between perception and action. Perception 36, 249–257 10.1068/p5449 17402666

[B40] Witt J. K. Proffitt D. R. Epstein W. (2005). Tool use affects perceived distance, but only when you intend to use it. J. Exp. Psychol. Hum. Percept. Perform. 31, 880–888 10.1037/0096-1523.31.5.880 16262485

[B41] Wölfflin H. (1886). Prolegomena to a psychology of architecture, in Empathy, Form and Space–Problems in German Aesthetics 1873–1893, ed Mallgrave H. F. (Santa Monica, CA: The Getty Center for the History of Art and the Humanities, 1994), 125–148

[B42] Wölfflin H. (1915). Principles of Art History. The Problem of the Development of Style in Later Art. New York, NY: Dover Publications (1932).

